# Spatial risk assessment of global change impacts on Swedish seagrass ecosystems

**DOI:** 10.1371/journal.pone.0225318

**Published:** 2020-01-24

**Authors:** Diana Perry, Linus Hammar, Hans W. Linderholm, Martin Gullström

**Affiliations:** 1 Seagrass Ecology and Physiology Research Group, Department of Ecology, Environment and Plant Sciences, Stockholm University, Stockholm, Sweden; 2 Department of Aquatic Resources, Swedish University of Agricultural Sciences, Lysekil, Sweden; 3 Octopus Ink Research & Analysis, Gothenburg, Sweden; 4 Regional Climate Group, Department of Earth Sciences, University of Gothenburg, Gothenburg, Sweden; University of Waikato, NEW ZEALAND

## Abstract

Improved knowledge on the risk in ecologically important habitats on a regional scale from multiple stressors is critical for managing functioning and resilient ecosystems. This risk assessment aimed to identify seagrass ecosystems in southern Sweden that will be exposed to a high degree of change from multiple global change stressors in mid- and end-of-century climate change conditions. Risk scores were calculated from the expected overlap of three stressors: sea surface temperature increases, ocean acidification and wind driven turbid conditions. Three high-risk regions were identified as areas likely to be exposed to a particularly high level of pressure from the global stressors by the end of the century. In these areas it can be expected that there will be a large degree of stressor change from the current conditions. Given the ecological importance of seagrass meadows for maintaining high biodiversity and a range of other ecosystem services, these risk zones should be given high priority for incorporation into management strategies, which can attempt to reduce controllable stressors in order to mitigate the consequences of some of the impending pressures and manage for maintained ecosystem resilience.

## Introduction

Human emissions of greenhouse gases to the atmosphere continue to rise [1]. This affects the ocean environment, not only through the resulting changes to atmospheric climate, but also through the oceans’ absorption of emitted CO_2_ [2]. Among alterations to the ocean environment caused by global change are increased sea surface temperature (SST), shifts in seawater carbonate chemistry, and changes in wind patterns, currents and salinity (reviewed by [3,4]). Such environmental alterations affect species differently and can be the cause of ecosystem state shifts, as dominant species may suffer deleterious effects while subordinate species are favored [5].

In temperate Swedish waters, global change is expected to have far-reaching consequences. The most dramatic anticipated changes are related to the freshening (desalination) of much of the Baltic Sea [6]. As a result of decreasing salinity the ranges of many Baltic species are expected to move southward, up to hundreds of kilometers, which can have far-reaching consequences for ecosystem functioning and species assemblages [7,8]. Shifts in seawater temperature and pH are also expected to have ecological consequences for the region. Research has even shown habitat-forming species, such as various macroalgae, and the regionally important blue mussel to be negatively influenced by the acidification [9,10], which can then cause cascading effects influencing many associated species. Resulting reductions in ecosystem resilience can make it more difficult for systems to recover from additional disturbance, as demonstrated by e.g. Eklöf et al. [11] in an experiment studying the effects of increased temperatures and ocean acidification on a simplified seagrass (*Zostera marina* L.) system. In fact, *Z*. *marina*, which has vanished on the Swedish west coast [12], has been shown to be unable to recover in areas where significant plant loss has in turn caused the resuspension of particulate matter into the water column, resulting in unfavorable conditions for the seagrass and, ultimately, a regime shift from seagrass meadows to unvegetated areas, even with restoration attempts [13].

Resuspension of particulate matter in the water column, causing turbidity, can occur as a result of wind-driven events [13]. These severe weather events, causing increases in wind, can also increase wave action in typically unexposed coastal areas. As a result of the wave action coastal habitats can suffer a lot of physical damage [14]. While such extreme events are typically short in duration, they are the cause of some of the largest amount of damage to ecological systems [15,16].

Global change stressors can, however, act in both a positive and negative manner depending on species and it has been shown that *Z*. *marina* is positively influenced by the oceans’ absorption of CO_2_, given that the plant is carbon-limited [17], and therefore ocean acidification in fact stimulates growth of *Z*. *marina* [18]. This increase in growth of plants has even been shown to offset the negative growth effects of increased temperature [19]. However, it has also been shown that while changes in seagrass biomass is where temperature stress is first seen, with an even greater increase in temperature, the plants ability to photosynthesize also decreases [20]. Conversely, evaluation of other trophic levels within the seagrass ecosystem has instead shown negative effects of global change related factors. For instance, shifts in the fish community within *Z*. *marina* meadows in Chesapeake Bay have been suggested as an effect of long-term increase in sea surface temperature [21]. Additionally, Alsterberg et al. [22] demonstrated experimentally the indirect consequences of climate change to the seagrass ecosystem. Their research showed that the removal of mesograzers in a *Z*. *marina* system exposed to increased sea temperature and acidified conditions led to an overgrowth of macroalgae and, therefore, a decrease in light availability negatively affecting benthic microalgae in the seagrass habitat. While specific species show different responses to various global change stressors, the evaluation of the combination of stressors within the whole ecosystem is what is both necessary and relevant for management purposes now and in the future.

The importance of evaluating the impacts of multiple stressors on marine species and ecosystems has been highlighted in recent years [4,23,24]. Although several stressors will coincide simultaneously at any one location as global change advances, the magnitude of exposure will differ geographically, even at local scales. The ecological risk posed by global change to individual habitats and species will, consequently, differ within and across seascapes. The ecological risk assessment (ERA) is a useful method for understanding the probability of adverse ecological effects caused by human-induced stressors [25]. Given that there can be a high degree of uncertainty in modeling data, the ERA has been considered a suitable method for dealing with such uncertainties [26], as it is a very straightforward analysis that does not require complicated calculation processes. Additionally, ERA has been deemed appropriate for evaluating risks at regional wide scales and for temporal variation [27]. Also, understanding the variation in risk for essential habitats may be a valuable contribution to management, as it facilitates geographical prioritization of conservation actions over time. This allows for the possibility of the designation of areas of high risk (“hotspots”) as well as ecological “refuges” where little threat is predicted [28].

Along the Swedish coasts, seagrass habitats are among the most important components of shallow-water ecosystems [29]. In essence, these habitats consist of seagrass growing in dense meadows on shallow mud or sand bottoms. Seagrass habitats provide multiple ecosystem services such as erosion reducing wave absorption, stabilization of sediment, carbon sequestration, as well as providing foraging grounds and shelter for a range of animals including fish and crustaceans [30–32].

In this paper, we take an ecological risk assessment approach and use existing spatial models to identify seagrass areas where three important global change related stressors during summer are likely to reach particularly high levels along the coastline of southern Sweden, for mid-century (2050) and end-of-century (2100). The stressors selected were (1) SST change, (2) ocean acidification, and (3) wind driven turbidity. The stressors were selected on the basis that they are expected to change in the region and expected to influence the seagrass coverage [11,22,33]. The aim of this study was to support management and identify potential risk regions for seagrass ecosystems along part of the Swedish coast under future climate change scenarios, by mapping areas of overlapping global change stressors based on differences from current conditions. The assessment indicates areas where seagrass ecosystems are likely to be exposed to comparatively high risks in a changing climate.

## Methods

### General method

Ecological risk assessment is the estimation of risk levels posed by human induced stressors to ecological receptors, with the purpose of supporting environmental management decisions [34]. We appoint the probability of seagrass habitat coverage, including seagrass plants and associated fauna, as the receptor for the current study. The three considered global change related stressors are increased summer sea surface temperature, ocean acidification, and summer wind-induced turbidity. The combination of these particular stressors has previously been shown to have a negative effect on seagrass ecosystems [33] and therefore the same combined stressors were used for this risk assessment. We do not include salinity change as a stressor because its combined effects with the other stressors of interest for this study were not previously evaluated and additionally, the most profound freshening of the Baltic Sea is expected to occur outside the geographical scope of the study, and its impacts are covered elsewhere [7,8].

Spatial representations of each considered stressor were developed from available Global Climate Models (GCMs) and regional climate models (described in more detail below) using the A1B greenhouse gas emission scenario [35–37] showing stressor levels on scales from 0 to 10, where 0 represents no change and 10 represents the highest expected change in the study region by the end of the 21^st^ century compared to current conditions. Risk scores were calculated as the spatial overlap of the three stressors, in areas with modeled occurrence of seagrass habitat. High-risk regions may be identified as areas with a high exposure to all three stressors in combination (*i*.*e*. an area with a high degree of exposure to only one stressor cannot be considered a risk zone in the current study).

### Designation of study region

**Fig 1:**
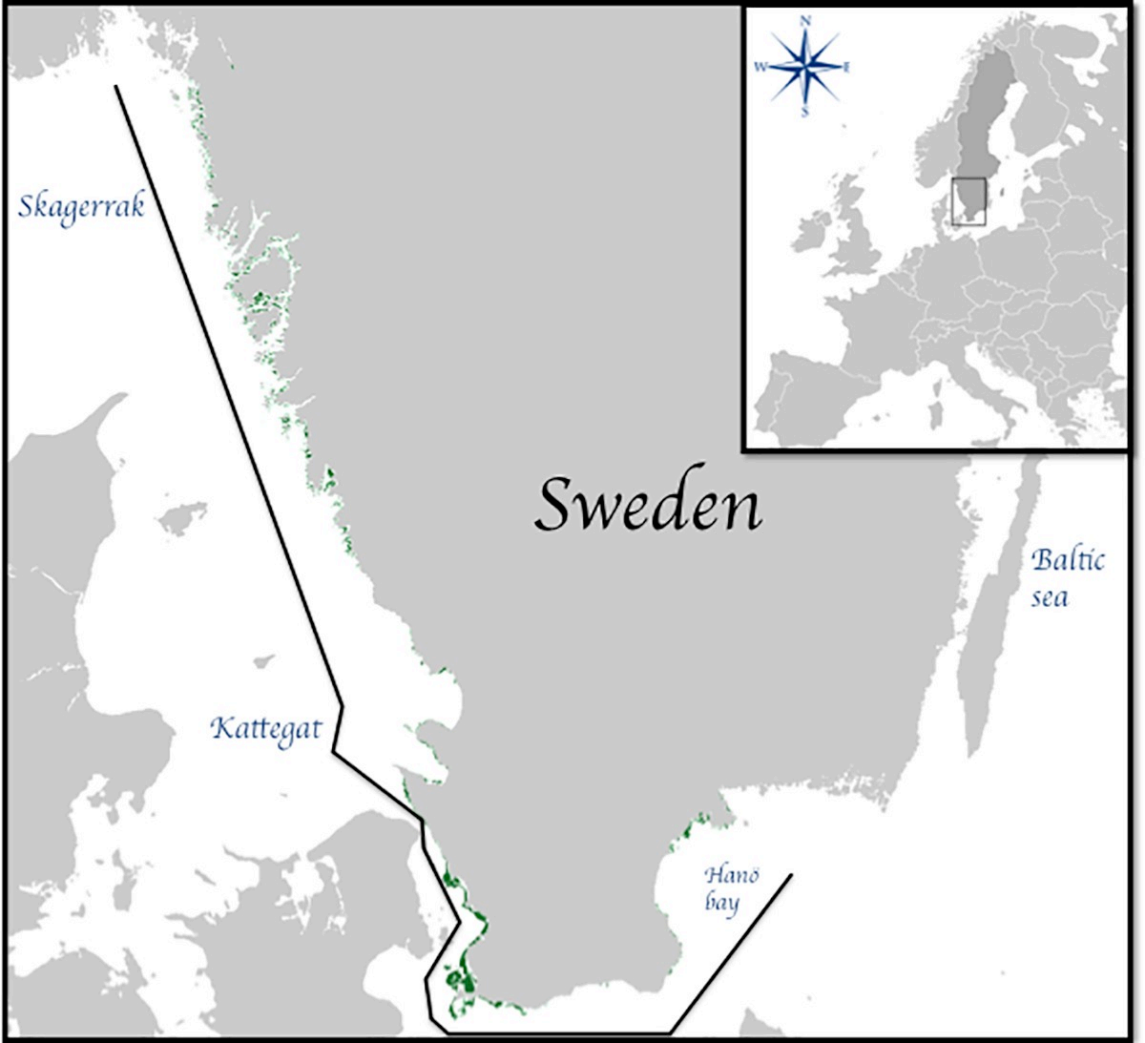
Map of the risk assessment study area (dark line). Green shades indicate modeled probability of marine angiosperm occurrence, used as proxy for seagrass (*Zostera marina*) habitat distribution. The model is for the current distribution of seagrass. Seagrass outside the study area is not shown. Coastline: Lantmäteriet.

Sweden has a long and diverse coastline stretching from the oceanic conditions in Skagerrak on the west coast, through the less saline Kattegat and into Öresund in the south, on to the brackish Baltic Sea and Bothnian Bay in the east and north. The seagrass distribution follows the salinity gradient with high occurrence along the western and southern coasts and vanishes north of Latitude 61° N in the Baltic Sea due to low salinity [38]. The salinity gradient within the Baltic plays a very important role in species distributions within the sea and Baden and Boström [39] suggest that seagrass within parts of the Baltic is at the limits of its salinity and temperature tolerance. Forecasted climate induced changes in Baltic Sea salinity indicate that the salinity threshold for seagrass and several associated species will move substantially southwards until 2100 [40]. This has played a role in why we have delimited our study area to the Swedish west coast (Skagerrak and Kattegat) and south coast (Öresund and the southern tip of the western Baltic Sea) 55–59°N to11-15°E, as indicated by Fig 1. It is important to note that the selected study area was chosen based on a couple of factors: a) the habitat distribution model is related to angiosperm cover (which includes freshwater green algae), and within the chosen study range this can be more reliably considered *Z*. *marina*, which is the focal habitat of this study [38] (there is a higher proportion of other angiosperm species within the lower salinity areas of the Baltic [41]), and b) salinity is not covered within the scope of stressors in the current study. Given the importance of salinity within the Baltic region, as well as the indication that seagrass within this area is already existing at its stress limits [39], we felt it most appropriate not to include this region in the risk assessment evaluation.

Within the study area, seagrass meadows composed of *Z*. *marina* occur on muddy and sandy bottoms from about 0.5 m down to approximately 6 m depth, where the main distribution ranges from 1 to 4 m depth [38]. Seagrass abundance has reduced significantly over the past decades due to eutrophication and physical disturbances, as well as the indirect cascading effects of fishing [42–44]. In addition to widespread seagrass meadows, the coastline is diverse with rocky archipelagos, estuaries and sandy beaches. Because of the geographical location, sheltered by the British Islands and Denmark, tides are insignificant in the area and the water level fluctuates mostly due to weather conditions [45].

### Receptor

An existing spatial model describing the probability of marine angiosperm occurrence was used as a proxy for the receptor, *i*.*e*. the probability of seagrass habitat coverage. The model, which is based on satellite remote sensing imaging (years 2008 and 2016) and validation by field observations (>600 sites mapped using conventional groundtruthing technique; [46]), is used by the Swedish Agency for Marine and Water Management as an indicator for seagrass coverage in the management tool “Symphony” for cumulative impact analysis in Sweden. Data and meta data are openly available [47]. The model describes probabilities of finding angiosperm stands in any 10 × 10 m pixel, aggregated into proportions of likely angiosperm occurrence in 250 × 250 m pixels (Fig 1). Data includes *Z*. *marina*, widgeon weed (*Ruppia* spp.), and occasionally some freshwater green algae species. Within the area of the current study, *Z*. *marina* constitutes the vast majority of angiosperms as the study area is marine, thus excluding freshwater species, and *Ruppia* spp. is found only occasionally along the inner margins of seagrass meadows [38]. The model covers the land-sea boarder along the coast and associated archipelagoes down to six meters depth, where angiosperm coverage can be accurately identified by satellite remote sensing. In this study, we considered areas where the angiosperm model predicts a ≥10% likelihood of occurrence for any given point within the 250 × 250 m pixel. This means that included areas are very likely to harbor some seagrass, although the coverage may not be very dense. Satellite remote sensing has been shown as an effective method for evaluating seagrass cover down to approximately 5 to 6 meters depth within the study area [46,48].

### Stressors

#### Temperature

**Fig 2:**
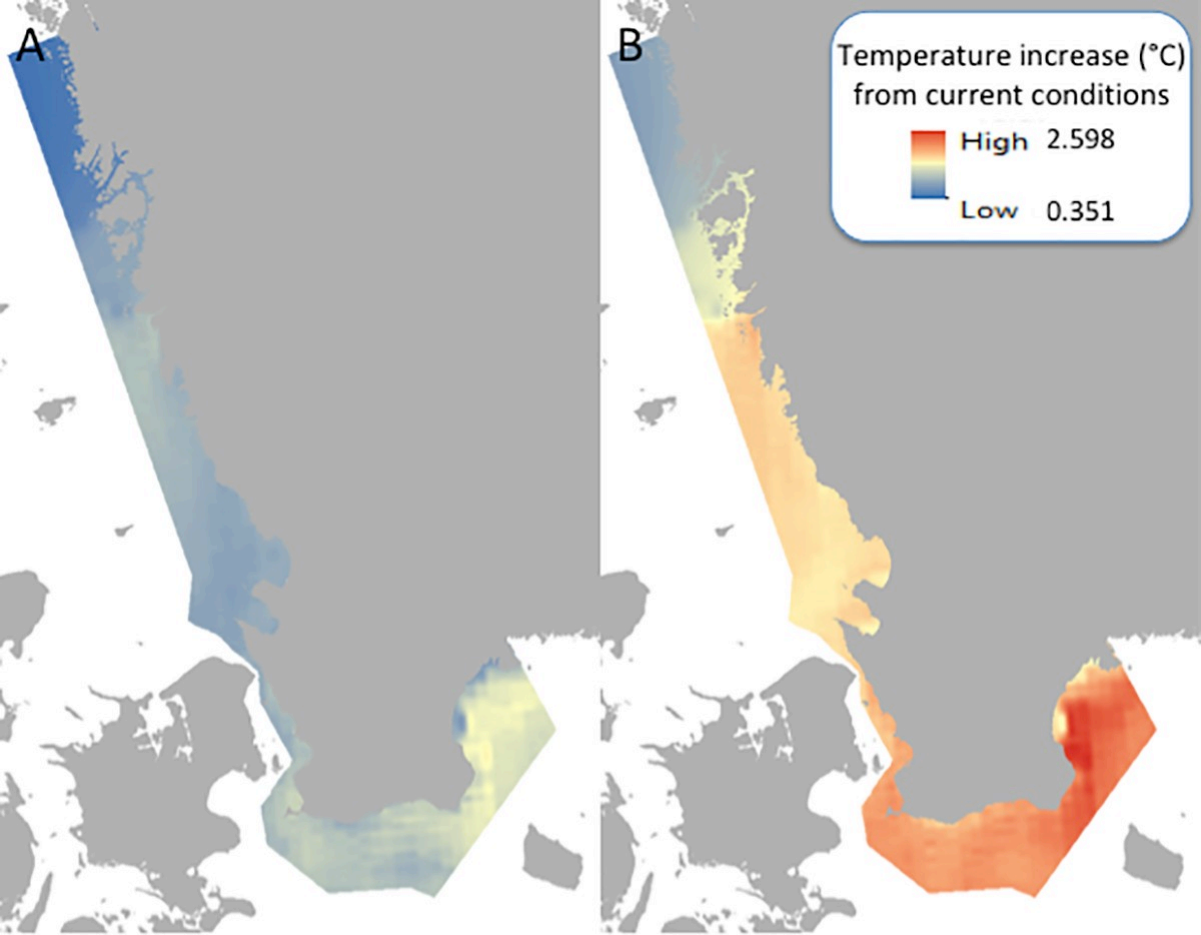
Differences in the modeled A1B scenario (from IPCC) on average summer sea surface temperature (°C) for A) mid- and B) end-of-century compared to current temperatures, shown as an increase in temperature. Coastline: Lantmäteriet.

Our basic assumption is that the intensity and duration of heat waves, which generate increased temperatures in shallow-water areas, are positively correlated with increasing SST as SST is averaged, and therefore would increase with the inclusion of increased temperatures in coastal areas [49]. We used existing spatial simulations (3.7 km grid) of the average summer (June-August) surface water temperature for the periods 2000–2029 (today), 2035–2064 (mid-century), and 2070–2099 (end of century) (Fig 2). The available data were pre-categorized into these time ranges. Simulations originate from the Swedish Meteorological and Hydrological Institute (SMHI) and are based on the Rossby Centre Atmosphere Ocean model (RCAO) coupled with the Swedish Coastal and Ocean Biogeochemical model (SCOBI). The simulations were forced by applying a dynamical downscaling approach using a regional climate model (RCM) with lateral boundary data from two General Circulation Models (GCMs), i.e. HadCM3 and ECHAM5/MPI-OM [35,50]. The emission scenario selected was based on the IPCC’s A1B scenario (high global emissions) [35,51], which was chosen given that shallow-water habitats are relatively adapted to variable conditions and therefore an evaluation of a high degree of change was of most interest for the current study. SMHI has tested the agreement between modeled data and observed values and found the agreement to be satisfactory, with most biases falling within the natural range of variation [35,50,52,53]. The available data projections do not cover part of the study area in Skagerrak, and therefore we used spline interpolation (as specified below) to obtain approximations for this area.

For each pixel, we subtracted modeled average summer SST of today from the average SST of the respective future time periods (mid- and end-of-century), hence deriving two spatial data sets describing the projected temperature change at mid-century and at the end of the century. Finally, we normalized the data linearly to a scale from 0 to 10. On this scale, value 0 equals no change from today and 10 corresponds to a 2.6°C increase (the highest average summer temperature in the end of century simulations).

#### Ocean acidification

**Fig 3:**
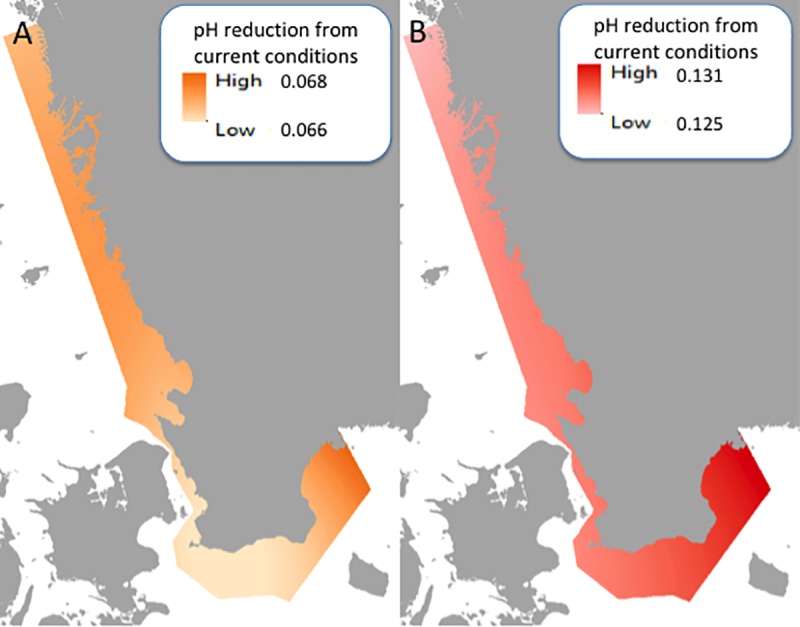
Differences in CMIP5 scenario projections for ocean pH for A) mid- and B) end-of-century compared to current pH values, shown as a decrease in pH. Coastline: Lantmäteriet.

For ocean acidification, we used spatial simulations of annual average seawater pH levels for 2016–2020 (today), 2046–2050 (mid-century), and 2096–2100 (end-of-century) (Fig 3). These data were obtained from the National Oceanic and Atmospheric Administration (NOAA) through the Climate Data Online database [36]. Given that these data were acquired from a different source than the temperature and wind data, slightly different time ranges were used, however they all cover the same ranges necessary for understanding current, mid- and end of century values. Once downloaded, the data coordinate system was converted for ArcGIS from tripole grid to a point data set, which was then interpolated to create raster files. The conversion was performed on the netCDF file using a Climate Data Operator [37,54,55]. The ocean acidification simulations are developed by NOAA for the CMIP5 projections, using global oceanographic models. Since this global data set has low spatial resolution (50 km grid) with partial gaps in the study area, we used spline interpolation (as specified below) to generate fully covering maps. We calculated the pH level differences between today and the two future scenarios and normalized the data linearly, as above, from 0 to 10. Value 0 indicates no change from today, while 10 refers to a pH decrease of 0.13 units.

#### Turbidity proxy

**Fig 4:**
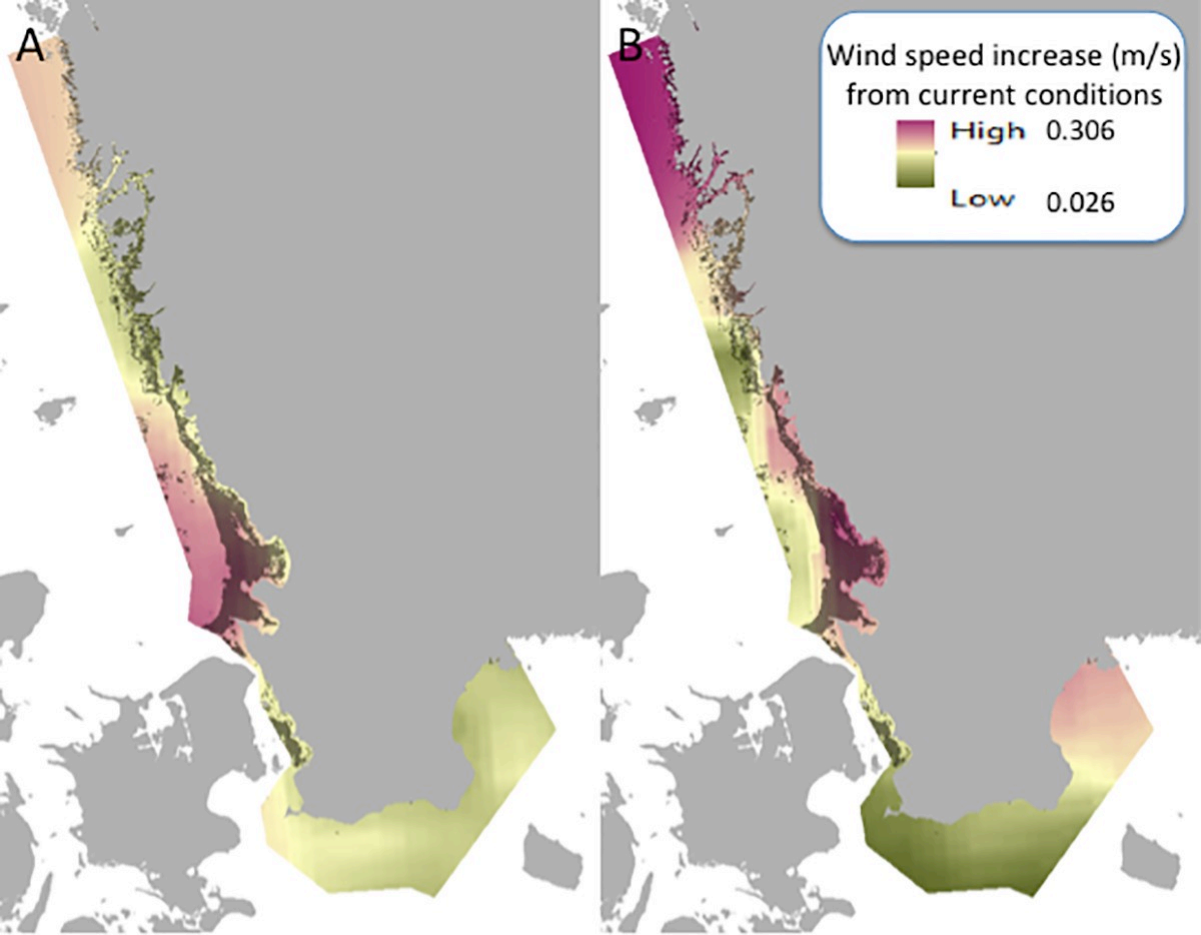
Differences in summer wind speed (m s^-1^) for A) mid- and B) end-of-century compared to current wind speed, shown as an increase in speed. This layer is combined with sediment data indicating soft bottom substrate in the photic zone, shown as dark shading. Coastline: Lantmäteriet.

Turbidity describes the amount of light-blocking particles in the water column. In shallow-water with no tides turbidity depends largely on sediment characteristics, depth and wind-driven wave action [13]. In this analysis, where only shallow waters (6 m or less) were considered, we roughly assume a direct positive correlation between average wind speed and the frequency of incidents with high turbidity, where sediments are fine grain and/ or muddy. We used existing simulations of average summertime (June-August) wind speed (m s^-1^, 10 m altitude; where wind speed calculation includes both mean wind = maximum ten minute mean wind over the last three hours, and wind gust = maximum two second mean wind over last 10 minute period;[35,50]), for the periods of 2000–2029 (today), 2035–2064 (mid-century), and 2070–2099 (end of century) (Fig 4). As for the temperature stressor, the simulations originate from the SMHI, and were developed through coupling of the RCAO and SCOBI models, with emissions based on the IPCC’s A1B scenario [35]. SMHI modeled wind speed data were validated using wind station observations [50]. Again, we used spline interpolation to attain approximations for Skagerrak, *i*.*e*. the northern west coast of Sweden.

Sediment type data were acquired from the Symphony tool [47], which was originally developed by the Geological Survey of Sweden. The sediment data indicate the likelihood of one out of three sediment classes in each pixel (250 × 250 m), based on substrate samples and depth data with relatively high accuracy along the coast and within the photic zone. Sediment classes are hard bottom (bedrock, rock and stone), transport bottom (sand and gravel) and soft bottom (e.g. mud, clay and silt). We only included areas with ≥25% likelihood of soft bottom substrate for any given point within the 250 × 250 m pixel.

Within these extracted (muddy) areas, we subtracted today’s wind speed from the wind speed of the two future scenarios. The obtained change in wind speed was normalized, with value 0 indicating no change and 10 indicating a 0.3 m s^-1^ average wind speed increase within shallow soft sediment areas, thus approximating likely locations of frequent turbid incidents.

### Calculation of risk

Risk scores were calculated as the sum of all three normalized stressors where these overlap with the receptor (stressor overlap in areas without seagrass coverage were excluded). In theory, this risk scale reaches from 0 to 30, where 0 means that none of the stressors change from today’s levels and a score of 30 indicates that all three stressors undergo the highest level of change at the same location, a location that also coincides with a likelihood of seagrass habitats. Since some stressors can generate varying effects when evaluated singly, negative effects on the receptor may only be granted where all three stressors coincide. Risk scores based on less than three stressors may not be considered high in the current risk assessment. Given the model uncertainties, only the highest combined scores should be further addressed. We suggest that, due to the screening characteristic of this assessment, only coherent areas with risk scores above 22.5 by the end of the century may be considered risk regions. Greater than 22.5 means >75% of the theoretical maximum of a combined change from current conditions. It also means that a significant change of all three stressors must be seen in order for the area to be considered under high risk. The precise level of this threshold is nevertheless arbitrary and only indicates that these areas are likely to be the most affected.

### Analysis tools

All data analyses were conducted in ArcMap v. 10.5, with data obtained in or converted to the geographic coordinate system WGS 1984. Interpolations were generated through the Spatial Analyst Tool using spline tension, weight 5, points 2. The reason for using a high weight and few points was to limit the influence of surrounding data points to only those in very close proximity.

### Uncertainties of assessments

This work has been derived from both downscaled global models and regional projections. Due to the diversity of data sources and formats, we could not calculate any quantitative measures of assessment uncertainty. Given the uncertainties in each of the combined data layers as well as the uncertainty of the underlying climate models, the accumulated uncertainty in the risk assessment is to be considered high. Furthermore, the assumption of a negative cumulative effect from the three selected stressors was based on literature, essentially the results acquired from a laboratory study [33], however negative effects from two stressors, temperature and OA, have also been shown to be negative for the *Z*. *marina* system [11,22]. It should be noted that the laboratory study by Perry et al. [33] evaluated effects of heat shock, turbidity and ocean acidification stress in combination (single stressor occurrence showed variable results with positive, negative or neutral effects, while in combination the outcome was always deleterious). However, no climate projection data is available for turbidity for the study region and thus a proxy for this stressor had to be used (*i*.*e*. wind speed plus soft sediment). The indirect relationship between the used versus the tested measure of turbidity incidents also adds to the uncertainty. While the risk assessment treats all three stressors equally in terms of contribution to the combined deleterious effect on the seagrass ecosystem, each stressor singly may not have an equal effect on the system, i.e. a high degree of change in OA might not be equally deleterious to a high degree of change in turbidity. However, it is the combination of these stressors that is of value for the current risk assessment.

As such, the identified risk regions should be evaluated with these limitations in mind. However, despite the limitations, it should not be discredited that there is an indication that some areas along the Swedish west coast are likely to be exposed to a high degree of change from the combined stressors over the course of the next century. This information is fundamental to the discussion of management of these important ecosystems in Swedish coastal waters as well as for projections in similar environments elsewhere.

## Results

The risk assessment for mid- and end-of century were calculated using differences between the current values for the three stressors, including SST, ocean acidification, and summer wind speed in soft sediment areas in the photic zone compared to the projected future values. The current stressor values for the seagrass areas within our study region showed a pH ranging from 7.79 in the Baltic to 7.90 in Skagerrak (SMHI modeled data tested against the control period showed agreement between modeled results and observations and was within the natural variation; [35]. Current summer sea surface temperatures range from 12.57 to 18.49°C, where the lowest temperatures are seen in southern Sweden and northern Bohuslän, whereas the highest temperatures are found in Kattegat. For the current summertime wind speeds the range is from 2.29 to 4.02 m s^-1^, with the highest wind speeds typically found farther offshore, while lower speeds can be seen closer to the coastline. The areas of low and high values showed similar patterns through time for the three stressors, however the range of low to high values varied with expected climate change. For mid-century, the pH decreased and ranged from 7.72 to 7.84, the sea surface temperature increased to a range of 13.71–19.32°C, and the wind speed increased from 2.40 to 4.12 m s^-1^. For the end-of-century values, the pH dropped to 7.66–7.78, the temperature increased from 14.50 to 19.99°C, while the wind speed ranged from 2.49 to 4.07 m s ^-1^.

### Risk assessment

#### Mid-century

**Fig 5:**
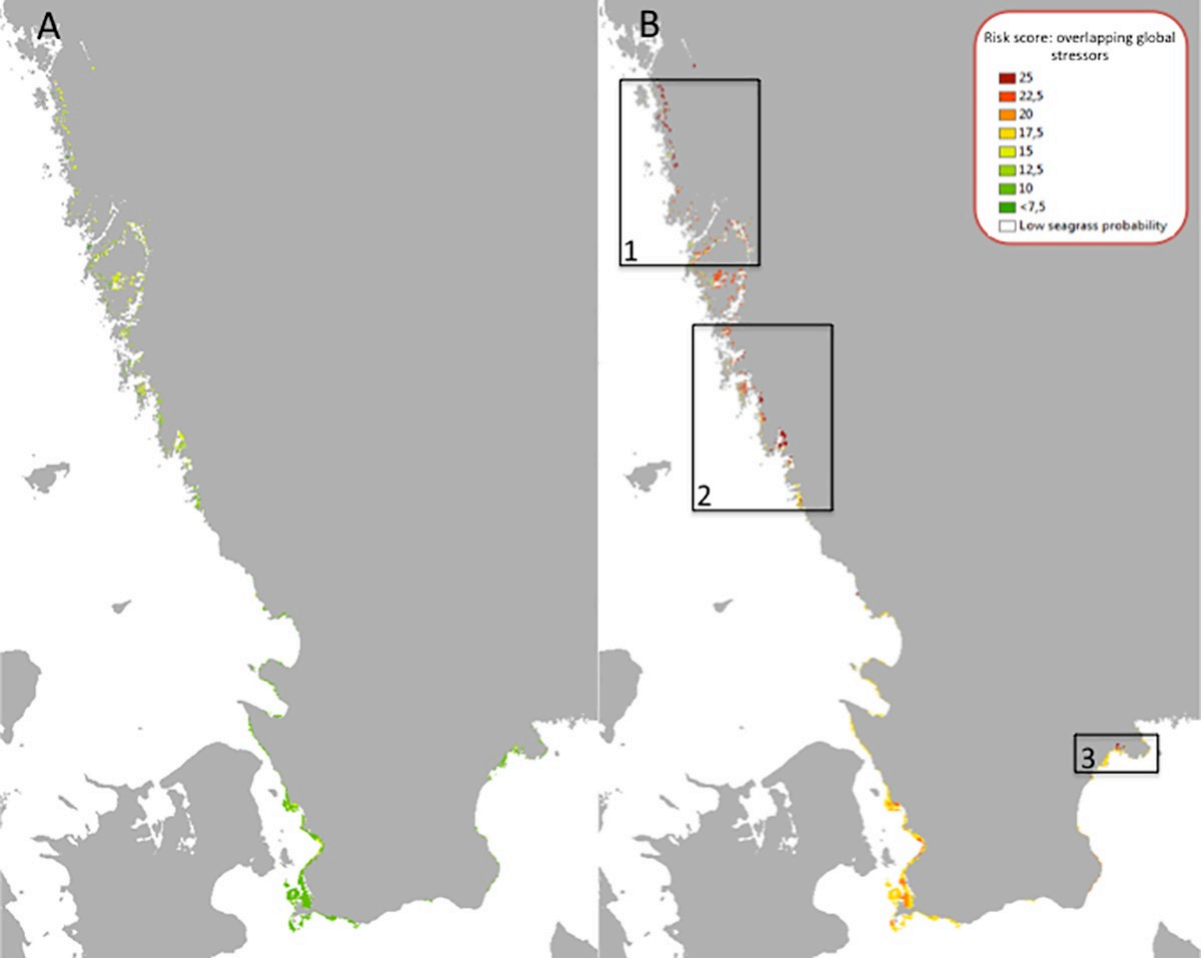
Risk assessment results at mid-century (A) and end-of-century (B) for seagrass cover in response to three global change stressors: summer sea surface temperature, ocean acidification, and summer wind speed in soft sediment areas. Labelled black boxes correspond to the zoomed images (panels 1–3) in Fig 6, showing regions of particularly high-risk scores. Areas with risk scores >22.5 can be considered risk regions. Coastline: Lantmäteriet.

For the mid-century period, around year 2050, the highest combined risk score was 14.8 (of a maximum 30), while the average was 10.1 ± 1.97 SD. The minimum risk score was 6.618. Given the relatively low risk scores and the high natural geographical variation in temperature and wind speed, there are no indications that seagrass habitats within the studied region will be at risk by the mid-century (Fig 5).

#### End-of-century

**Fig 6:**
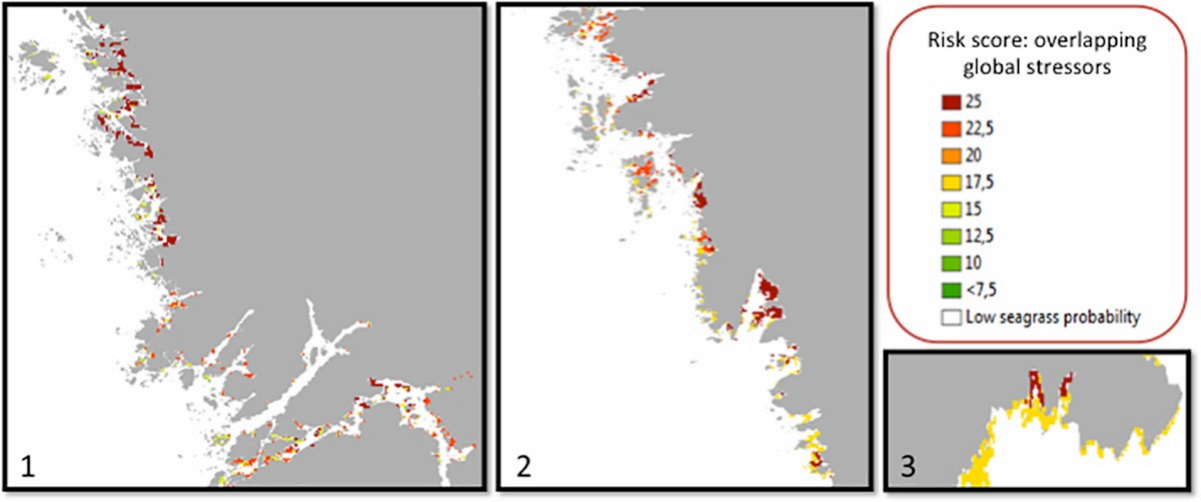
Locations with identified risk regions for the end-of-century time period, where areas in red correspond to the highest level of combined (coinciding) stressors (sea surface temperature, ocean acidification and wind- driven turbidity) within the seagrass ecosystems. Coastline: Lantmäteriet.

For the end-of-century period, around year 2100, resulting risk scores range from 12.7 SD to 25.0 with an average of 18.4 ± 2.75 SD within seagrass habitat areas. Risk regions, defined as areas with risk scores above 22.5, appear in three different regions in northern and southern Skagerrak as well as in the Hanö Bay in the Baltic Sea (Figs 5 and 6).

## Discussion

Most strikingly, the results of the current assessment indicate three different regions in Sweden where seagrass habitats may be at particular risk from combined global change stressors by the end of the 21^st^ century. In these risk regions, it may be expected that seagrass will be exposed to multiple overlapping stresses from increased sea surface temperature, increased ocean acidification and increased wind speed that causes turbid water conditions. The identified risk regions are inshore northern Skagerrak (Fig 6, panel 1), inshore northern Kattegat (Fig 6, panel 2) and the northeast corner of the Hanö Bay in the Baltic Sea (Fig 6, panel 3). While the mid-century results in many places show elevated values for the stressors involved, the combined effect may still be relatively low according to this assessment. The future projections indicate that the overall geographical patterns will prevail, despite shifts in averages and ranges. Only for pH levels are the anticipated temporal changes due to global change clearly exceeding the spatial variations of today within the study area. Both temperature and wind speed will shift by means and ranges, but without much change from the spatial variation of today.

Interestingly, for the end of the century, the results indicate that risk regions are more apparent in sheltered areas, whereas more exposed locations along the coast typically have lower risk scores. In the first identified and largest risk region, i.e. inshore northern Skagerrak (Fig 6, panel 1), the area shows a low pH combined with an increase in wind speed and abundant soft sediments in shallow areas, though a temperature change seems to contribute to a lesser degree. These patterns may be due to the fact that in sheltered areas there is often less wave action due to protective land barriers, which therefore leave water conditions more stagnant, making increases in temperature and ocean acidification more acute in these locations. In fact, Pihl et al. [56] discuss the importance of wave exposure and water exchange along the Swedish coast.

The second identified risk region is the inshore northern part of Kattegat (Fig 6, panel 2). Here, the expected increased wind speed is slightly lower than in northern Skagerrak, but the combined risk scores are still high as a result from larger shifts in temperature and pH. In northern Kattegat, high-risk areas for seagrass habitats are of smaller size and more disjointed in spatial extent than in the northern Skagerrak because inshore, muddy bottoms, which are likely to generate turbid water during strong winds, are less common in this sand- and rock-dominated area. Notably, however, seagrass restoration efforts in parts of northern Kattegat/southern Skagerrak have shown strong significant effects of wind in the area causing turbid conditions that have, in some cases, created a regime shift from previously established seagrass meadows to unvegetated areas where the natural recovery of seagrass is prevented [13], with smaller meadows less able to dampen the impacts of strong waves making individual shoots potentially more susceptible to damage/destruction from strong wave action. Therefore, while the current study shows that the changes in wind expected in the future are less than that found in Skagerrak, the seagrass in this area appears to be particularly sensitive to any increases in wind already making any future intensifications even more impactful.

The third identified risk region (Fig 6, panel 3) is spatially rather confined in comparison to the other risk regions as it is located in the northern part of the Hanö Bay in the Baltic Sea. This area may be subjected to particularly high changes in temperature and pH with future global stressors. The reason for its high risk is the large amount of muddy sediment, which differs from the surrounding sand bottom areas.

Notably, no risk regions are identified along the rather exposed Kattegat coastline. The lack of risk regions in southern Kattegat, and into parts of the Baltic Sea may be dependent on either the absence of seagrass, or the low abundance of muddy sediments (sandy sediments dominate). Projections of temperature and pH, however, indicate expected changes in the area in the future. Interestingly, the spatial variation of ocean acidification is small across the studied area, but the temporal change, with substantial pH decrease throughout the region, contributes strongly to risk at most locations by the end-of-century. Low pH is, therefore, a driver for elevated risk in all areas in the later future scenario, while it does not contribute much in the mid-century scenario. Along the ecologically important coastlines of California, high temporal- but low spatial variability has also recently been shown leading the authors to suggest that there may be some local species adaptability possible in persistently low pH areas over time [57].

Using the terminology of Queiros et al. [28], the high-risk regions identified through the current assessment can be considered “hotspots” given that they show high ecosystem vulnerability, whereas the seagrass meadows in parts of Kattegat and Öresund may instead be thought of as “refuges”, or areas with low ecosystem vulnerability. This concept may hold high potential for use in Swedish coastal management in the future. Interestingly, the same area here appointed as a risk zone in the Hanö Bay (Fig 6, panel 3), has previously been considered a possible climate refuge due to the increasing desalination of the Baltic, leaving this area one of the last remaining locations for Baltic seagrass meadows by the end of the century [8] However, if the current findings hold, the conclusion may be that this particular area is, in fact, suboptimal as a refuge when multiple climate factors are accounted for simultaneously.

The identified risk regions should be of particular concern given that the seagrass meadows in Sweden are already a stressed system faced with eutrophication and overfishing [12,43,44,58]. Notably, research has illustrated that temperatures above 25ºC are considered stressful for *Z*. *marina* [59,60] and with an increase in average SST of up to 20°C the likelihood of heat wave events causing temperatures to surpass 25°C also potentially increases. Additionally, *Z*. *marina* beds in the area have been shown to be a very dynamic system showing shifts in coverage and site locations over years [61]. Stressed ecosystems are less resilient to pressures, show a loss in biodiversity, and a subsequent decrease in ecosystem functioning [62], making ecosystem state shifts more likely. Biodiversity loss, in turn, creates ecosystems less resilient to multiple stressors [63]. Due to the sediment trapping properties of seagrass, a loss in coverage can lead to the resuspension of particulate matter into the water column, which in turn creates turbid conditions that make settlement and recolonization of seagrass much more difficult [31]. This may create a negative feedback loop, where loss of plants leads to turbid water causing unfavorable conditions for seagrass and potentially further losses [13].

Given the high productivity of the *Z*. *marina* ecosystem on the Swedish coast ([40], Deyanova et al unpublished), threats to the system should be taken seriously. A reduction in the area of seagrass meadows can cause far-reaching ecological consequences for many species, including economically valuable fisheries species such as cod [43,64]. This particular species can be thought of as an example, given that it is a very important fish species within seagrass meadows in Sweden, and therefore declines in cod numbers have the potential to threaten other shallow-water coastal habitats. Due to its high mobility and use of large areas, the species connects many habitats throughout its lifetime, both within the shallow-water coastal areas and across nearshore-offshore seascapes [65–68].

### Limitations

While the results of the current study give valuable indications for future climate change consequences by the end of the century in seagrass ecosystems along the Swedish coast, it is important that the results are interpreted with a degree of caution. Given that the risk assessment is based on climate scenarios from different model simulations (NOAA and SMHI) and therefore slightly different model forcing methods were used and the time ranges were not precisely the same, the results contain uncertainties. However, the data used is quality data, which has been tested against true observations and was the best available though future projections are inherently uncertain. As well as model data uncertainties, the additive-type analysis was somewhat simplistic in nature and not without its own limitations and because of the restrictions in the availability of data, proxies for risk-related stressors were used for the current study. However, while it is prudent to keep in mind these limitations, the study is a first synthesis of its kind along the Swedish coast, and until updated data are available the results may be valuable for the management of seagrass ecosystems in Sweden in the face of future climate change and a similar methodology employed for evaluating seagrass ecosystems in other areas where such data are available.

The current risk assessment is of a simplistic nature and should be updated as newer climate simulation data become available; however, in the meantime, the results may serve as a valuable contribution to management discussions. Given the high ecological value of *Z*. *marina* beds in maintaining coastal biodiversity, understanding future threats to the ecosystem allows for the possibility of managing the areas in such a way as to increase the chances of preserving high ecosystem functioning despite the impending changes. As Havenhand and Dahlgren [6] suggest, results such as these should be considered in marine spatial planning strategies, in order to best manage the valuable ecosystems of today, for mitigating the impacts of the future.
